# Case Series of Myocarditis Following mRNA COVID Vaccine Compared to Pediatric Multisystem Inflammatory Syndrome: Multicenter Retrospective Study

**DOI:** 10.3390/vaccines10081207

**Published:** 2022-07-29

**Authors:** Yonatan Butbul Aviel, Philip J. Hashkes, Yotam Dizitzer, Kanteman Inbar, Yackov Berkun, Eli M. Eisenstein, Mohamad Hamad Saied, Ofra Goldzweig, Merav Heshin-Bekenstein, Eduard Ling, Michal Feldon, Rotem Tal, Shiran Pinchevski-Kadir, Irit Tirosh, Liora Harel, Gil Amarilyo, Kfir Kaidar

**Affiliations:** 1 Department of Pediatrics and Pediatric Rheumatology Service, Rambam Health Care Campus, Ruth Children’s Hospital, Haifa 2611001, Israel; inbar.huberman@gmail.com; 2 Pediatric Rheumatology Unit, Shaare Zedek Medical Center, Hebrew University-Hadassah School of Medicine, Jerusalem 9103401, Israel; hashkesp@szmc.org.il; 3 Schneider Children’s Medical Center, Sackler School of Medicine Tel-Aviv University, Tel Aviv 6329302, Israel; yotamdiz@gmail.com (Y.D.); rotemtal77@gmail.com (R.T.); liorahar@clalit.org.il (L.H.); gamarilyo@clalit.org.il (G.A.); kfir.kaidar@clalit.org.il (K.K.); 4 Department of Pediatrics, Hadassah-Hebrew University Medical Center, Mount Scopus, Jerusalem 9103401, Israel; berkun@hadassah.org.il (Y.B.); emeisenstein@gmail.com (E.M.E.); 5 Carmel Medical Center, Technion–Israel Institute of Technology, Haifa 2611001, Israel; mohamadhs@clalit.org.il; 6 Kaplan Medical Center, Rehovot, Hebrew University and Hadassa, Jerusalem 9103401, Israel; ofragol@clalit.org.il; 7 Pediatric Rheumatology Service, Dana-Dwek Children’s Hospital of Tel Aviv Medical Center, Sackler School of Medicine, Tel Aviv University, Tel Aviv 6329302, Israel; meravheshin@gmail.com; 8 Saban Pediatric Medical Center for Israel, Pediatrics Department B, Soroka University Medical Center, Beer Sheva 84417, Israel; eduardling@bgu.ac.il; 9 Shamir Medical Center, Sackler School of Medicine, Tel-Aviv University, Tel Aviv 6329302, Israel; michal@feldon.com; 10 Sheba Medical Centre, Tel Hashomer, Sackler Faculty of Medicine, Paediatric Rheumatology Unit and Department of Paediatrics B, Edmond and Lily Safra Children’s Hospital, Tel-Aviv University, Tel-Aviv 6329302, Israel; shiranpinch@gmail.com (S.P.-K.); irit.tirosh@gmail.com (I.T.)

**Keywords:** myocarditis, COVID vaccine, PIMS

## Abstract

Introduction: Since the development of COVID-19 vaccines, more than 4.8 billion people have been immunized worldwide. Soon after vaccinations were initiated, reports on cases of myocarditis following the second vaccine dose emerged. This study aimed to report our experience with adolescent and young adults who developed post-COVID-19 vaccine myocarditis and to compare these patients to a cohort of patients who acquired pediatric inflammatory multisystem syndrome (PIMS/PIMS-TS) post-COVID-19 infection. Methods: We collected reported cases of patients who developed myocarditis following COVID-19 vaccination (Pfizer mRNA BNT162b2) from all pediatric rheumatology centers in Israel and compared them to a cohort of patients with PIMS. Results: Nine patients with post-vaccination myocarditis were identified and compared to 78 patients diagnosed with PIMS. All patients with post-vaccination myocarditis were males who developed symptoms following their second dose of the vaccine. Patients with post-vaccination myocarditis had a shorter duration of stay in the hospital (mean 4.4 ± 1.9 vs. 8.7 ± 4.7 days) and less myocardial dysfunction (11.1% vs. 61.5%), and all had excellent outcomes as compared to the chronic changes among 9.2% of the patients with PIMS. Conclusion: The clinical course of vaccine-associated myocarditis appears favorable, with resolution of the symptoms in all the patients in our cohort.

## 1. Introduction

Coronavirus disease 2019 (COVID-19) has affected more than 380 million people globally since it was declared a pandemic by the World Health Organization (WHO) on 11 March 2020 and has caused more than 5.7 million deaths [1].

In April 2020, the first pediatric patient with severe multisystem disease following COVID-19 infection was described in the UK [2]. Since then, many countries have reported very sick children presenting with features of substantial inflammation temporally related to COVID-19 [3]. Symptoms usually begin a few weeks following infection and include features of incomplete and atypical Kawasaki disease (with or without coronary artery dilatation), toxic shock syndrome, and myocarditis with impaired cardiac function [4]. Preliminary case definitions of this novel inflammatory condition have been published by the UK Royal College of Paediatrics and Child Health (RCPCH) [5], the US Center for Disease Control and Prevention (CDC) [6], and the WHO [7]. The CDC and WHO jointly named the condition “multisystem inflammatory syndrome in children” (MIS-C), and it is also known as pediatric inflammatory multisystem syndrome (PIMS/PIMS-TS).

In October 2020, the first successful results from phase 1 clinical trials of the Pfizer vaccine candidate BNT162b2 were published. The vaccine is a nucleoside-modified RNA (mRNA) encoding the SARS-CoV-2 full-length spike, modified by two proline mutations to lock it in the prefusion conformation [8]. The study was conducted on 43,548 volunteers 16 years of age or older, randomized in a 1:1 ratio; 21,720 people received the vaccine. A few months later, the data on the safety and efficacy of the BNT162b2 mRNA COVID-19 vaccine were published [9].

The BNT162b2 vaccine is 95% effective in preventing COVID-19, and its safety profile is characterized by short-term, mild-to-moderate pain at the injection site; fatigue; and headache. The incidence of serious adverse events was low and similar in both the vaccine and placebo groups.

Recently, a study that evaluated the efficacy and safety of the same vaccine among 1131 adolescent children aged 12–15 years was published. While the vaccine showed good efficacy and was safe, more patients reported adverse effects that ranged from mild to moderate, mostly local reaction or fever, and only 0.6% reported severe adverse effects, with no cases of myocarditis reported [10].

Israel was one of the first countries in the world whose residents were widely vaccinated with the BNT162b2 vaccine. In April 2021, the first report on the effectiveness of vaccines in a nationwide mass vaccination setting was published by the largest Israeli health care organization. The study group included 596,618 persons older than 16 years and the vaccines were proved to be very effective, reducing the number of documented infections, symptoms, hospitalizations, and cases of mortality from COVID-19 [11].

A short time after the beginning of mass vaccinations, a few pediatric centers in Israel reported cases of myocarditis in adolescents following the first or second vaccine dose [12]. The aim of our study was to characterize those cases and compare them to our experience with patients with PIMS who developed myocarditis following infection by COVID-19.

## 2. Materials and Methods

In April 2021, subsequent to the first reports of myocarditis following COVID-19 vaccinations, we contacted all pediatric rheumatology centers in Israel (11 centers) and asked them to report all myocarditis cases assumed to have occurred following COVID-19 vaccination.

Patients that were included in the study were 16–21 year old individuals who developed myocarditis following COVID-19 vaccination (Pfizer mRNA BNT162b2) within 2 months after their first or second vaccine dose. Myocarditis was defined by classical symptoms (chest pain, shortness of breath, palpitation, etc.) associated with either an abnormal cardiac enzyme (brain natriuretic peptide (BNP) or troponin) or abnormal cardiac function, as demonstrated by echocardiogram or magnetic resonance imaging (MRI) of the heart.

During the study period, COVID vaccination was only approved for patients older than 16 years in order to better define the differences between patients with PIMS and patients with myocarditis following COVID-19 vaccination. We compared patients with PIMS who were older than 16 years to patients with younger ages at presentation.

Patients who developed myocarditis followed COVID-19 vaccination (Pfizer mRNA BNT162b2) were compared to patients with PIMS diagnosed in the pediatric rheumatology centers in Israel between April 2020 and April 2021. Cases of PIMS were defined according to the case definition published by the UK RCPCH [7], CDC [8], and WHO.

## 3. Statistical Analysis

All statistical analyses were performed using SPSS statistical package (SPSS, Chicago, IL, USA). All data are expressed as medians, means ± standard deviation, or as percentages. The Chi-squared test, Mann–Whitney U-test, and Student’s *t*-test were used, and a *p*-value lower than 0.05 was accepted as statistically significant. Each hospital’s local Helsinki Committee approved the study.

## 4. Results

### 4.1. Myocarditis Post-Vaccine Group

We identified nine patients from four centers who all developed symptoms of myocarditis following their second vaccine dose for COVID 19. Clinical and epidemiological information, including background diseases, are presented in Table 1. Two patients had prior myocarditis—one year and a few years previously—and both had complete resolution of the first event.

All the reported cases were males. Although more than half were admitted to a pediatric intensive care unit (PICU), most had only mild-to-moderate disease. Only one required inotropic support, and none required mechanical ventilation. The mean stay in the PICU was 3.6 ± 1.3 days (median: 2 days) and the mean total hospital stay was 4.4 ± 1.9 days (median: 4 days).

Four patients reported fevers higher than 38 °C upon admission; all reported chest pain, and three reported shortness of breath.

All patients except one had normal ejection fractions (EFs) (64% ± 29) upon admission; four patients showed abnormal findings in echocardiograms, all of which were mild (one with mild aortic insufficiency, one with mild mitral and tricuspid regurgitation, one with mild tricuspid regurgitation, and one with posterior wall dyskinesia). None of the patients showed signs of pericarditis. All patients had normal heart function at their follow-up echocardiogram (20 ± 14 days post-discharge).

One patient had an MRI of the heart 2 months post-discharge, showing mild basal hypokinesia in the lower part of left ventricle with mild subepicardial edema consistent with recent myocarditis.

All patients except one had elevated levels of troponin on admission. The patient with normal levels of troponin at presentation later developed increased levels. Only two of the nine patients had elevated CRP levels during their stays in hospital.

### 4.2. MIS-C Group and Comparison to Myocarditis Groups

We identified 78 patients from nine centers in Israel with a diagnosis of PIMS and compared their clinical and demographic characteristics to those of patients with post-vaccination myocarditis (Table 2).

Patients in the post-vaccination myocarditis group were older. No patients in the post-vaccination myocarditis group fulfilled the criteria for Kawasaki disease, although two had some features of Kawasaki disease (patients 7 and 8); both had mucosal changes, and patient 7 also had lymphadenopathy. In the PIMS group, 11.5% of patients fulfilled the criteria for Kawasaki disease and 84.6% had at least one feature of Kawasaki disease.

Patients in the post-vaccination myocarditis group had a milder disease, usually requiring no therapy or only short-term supportive therapy, such as NSAIDs. Although 66.6% of them were admitted to ICU, most required only a short stay in the ICU, as compared to 25.6% of patients in the PIMS group who required mechanical ventilation; no patients in the myocarditis group required such support.

Nine patients (11.5%) with PIMS were older than 16 years old. Compared to younger patients with PIMS, those patients had more severe courses with longer stays in the PICU and required respiratory support more often (Table 3).

## 5. Discussion

Recent studies have shown that the two-dose BNT162b2 vaccine is safe and 95% effective against COVID-19. The vaccine was initially studied in adults older than 16 years and later proven to be safe and effective in adolescents between 5 and 16 years of age [10].

The reported adverse effects of the COVID-19 vaccine are mild overall; systemic adverse effects were reported more often by younger vaccine recipients (16 to 55 years old) than older vaccine recipients and more often after the second dose. The frequency of any severe systemic event after the first dose was less than 1%, with severe systemic events reported in less than 2% of vaccine recipients after either dose [9].

In a study evaluating 2260 12–15 year old adolescents who received similar vaccines against COVID-19 (1129 placebos and 1131 who received the vaccine), 50–60% of vaccinated patients reported mild systematic adverse effects (mainly fatigue and headache with no serious events), with systemic events reported more often after the second vaccine dose [10].

Initial reports of myocarditis post-vaccination have emerged predominantly among males in the Israeli army after their second dose of the mRNA vaccine [13].

Recently, an Israeli study by Mevorah et al. reviewed data from medical records obtained from the Israeli Ministry of Health regarding hospitalized patients with suspected myocarditis among vaccinated persons as compared to unvaccinated controls during the 6 month period from December 2020 through May 2020. The results showed that there were 136 definite or probable cases of post-vaccinal myocarditis reported during the surveillance of more than 5 million vaccinated persons; 129 cases were reported to be mild and one fulminant case was fatal. The overall risk difference between the first and second doses was 1.76 per 100,000 persons, with the largest difference being among male recipients between the ages of 16 and 19 years. The rate ratio 30 days after the second vaccine dose in fully vaccinated recipients, as compared to unvaccinated persons, was 2.35 (95% CI: 1.10 to 5.02) [14].

Another study from Israel by Witberg assessed 2.5 million vaccinated persons listed in the database of Clalit Health Services and identified 54 cases that fulfilled the definition of myocarditis; all the patients had a favorable outcome. The estimated incidence of myocarditis up to 42 days after at least one dose of vaccine was 2.13 cases per 100,000 persons, with the highest incidence being among young male [15]. The findings in both studies were similar to our findings of higher myocarditis following the second dose of the vaccine, higher occurrence among males, and overall favorable outcomes.

Audrey et al. reported their experiences with 15 adolescents who developed myocarditis post-vaccination. Similar to our study, 93% of cases were male, all developed symptoms following their second dose, and most patients had normal myocardial function, with none admitted to the ICU [16]. Although most of our patients had mild disease, many were still admitted to the ICU. We assume this was related to uncertainty regarding the severity of the symptoms.

The estimated incidence for myocarditis post-mRNA vaccine is 0.008% for adolescents and 0.01% in children aged 12–15 years. Almost all the reported cases occurred following the second vaccination dose; thus, one speculation is that there is a hyper-immune response to the second dose [12]. Children with PIMS demonstrate a more robust immune response to SARS-CoV-2 infection than adults with COVID-10 infection, suggesting it may be beneficial to decrease the second vaccine dose or increase intervals between the doses. The efficacy and safety of such a policy should be further evaluated.

Although all patients with post-COVID-19 vaccine myocarditis recovered completely in our series, some recent cardiovascular MR findings indicate that there might be potential for late myocardial fibrosis, and the long-term impact of post-vaccine myocarditis remains unknown [17].

Our series demonstrated favorable outcomes for post-vaccine myocarditis compared to patients with PIMS. Patients with PIMS in our series were younger than the patients with post-vaccination myocarditis, and the differences were mainly due to the population that was vaccinated during the period of data collection (older than 16 years) and the greater tendency to develop myocarditis post-vaccination in adolescents and young adults than in younger patients [14]. When comparing PIMS in patients younger than 16 years and in patients who are 16 years old and above, younger patients tend to have a milder disease. These findings were previously reported for other PIMS series and further emphasize our findings concerning favorable outcomes for patients with post-vaccine myocarditis compared to patients with PIMS [18]. Another limitation of our study is that it was a multicenter study involving nine centers. We tried to overcome this limitation by using the same case definition (UK RCPCH [7], CDC [8], and WHO) and by collecting detailed information about clinical course treatments and outcomes.

It is noteworthy that two of our patients had previous bouts of myocarditis, and a previously published vaccine-associated myocarditis series showed a family history of first-degree relatives with myocarditis. This finding suggests a partial genetic susceptibility for myocarditis [12]. Therefore, we recommend that patients with a previous history or family history of myocarditis be closely monitored following the second dose of the COVID-19 vaccine.

The CDC Advisory Committee on Immunization Practices identified a likely association between the two COVID-19 mRNA vaccines from Pfizer-BioNTech and Moderna and cases of myocarditis and pericarditis [19]. According to this committee, there were 1226 reports of probable myocarditis/pericarditis cases in the vaccine Adverse Event Reporting System (VAERS) out of the approximately 300 million COVID-19 mRNA vaccine doses administered, 67% of which followed the second dose. Seventy-nine percent were in males, with the majority in individuals <30 years of age, with a median age of 24 years. Median time to onset of symptoms was 3 days after vaccination among patients 16–18 years old. Most patients were hospitalized for short periods and had good outcomes [19]. 

The reason for male predominance in post-vaccine myocarditis is still obscure, although there have been some speculations. In general, male predominance in myocarditis/pericarditis cases has been described in prior clinical and experimental studies [20], and, historically, post-vaccination myocarditis has been reported as a rare adverse event after vaccinations, especially after smallpox, influenza, and hepatitis B vaccinations. Interestingly, 79% of the reported cases were in males [21].

One of the explanations suggested is sex hormone differences. Testosterone is thought to play a role though a combined mechanism of inhibition of anti-inflammatory cells [3] and commitment to Th1-type immune response. Estrogen has inhibitory effects on proinflammatory T cells, resulting in a decrease in cell-mediated immune responses, and pericarditis incidence is higher in women during the postmenopausal period. Another contributing factor could be underdiagnosis in women [12].

Recently, on 2 November 2021, the Advisory Committee on Immunization Practices (ACIP) in the USA issued an interim recommendation for the use of the Pfizer-BioNTech COVID-19 vaccine in children aged 5–11 years for the prevention of COVID-19 [22]. Millions of young children have received the vaccine since then and there have been no reports of myocarditis among young children receiving the vaccine, which strengthens the theory that sex hormones play a role in the pathogenesis of post-vaccination myocarditis.

There are still no good data regarding mRNA COVID-19 vaccines for patients with a history of myocarditis or pericarditis after a dose of an mRNA COVID-19 vaccine, but the CDC recommends that precautions should be taken and subsequent doses of any COVID-19 vaccines should generally be avoided [23].

## 6. Conclusions

Despite these rare cases of myocarditis, the benefit–risk assessment for COVID-19 vaccination shows a favorable balance for all age and sex groups. Given the known potential risk of complications from COVID-19 infection, including hospitalizations, PIMS, and even death in young adults, as well as prolonged post-COVID-10 symptoms in many patients, the risk–benefit decision remains favorable for vaccination. Therefore, COVID-19 vaccination is currently recommended for every person older than 5 years of age. Caution is advised for patients with prior myocarditis but, according to a few national recommendations, this should not be a contraindication for COVID-19 vaccination. Nevertheless, caution is suggested, especially if pericarditis or myocarditis has occurred in the last 3 months priors to vaccination [24].

## Figures and Tables

**Table 1:** Clinical and epidemiological data for nine patients presenting with myocarditis following COVID-19 vaccine.

Patient No.	Age (yrs)	Gender	Days from Second Dose	Background Diseases	Chest Pain	Dyspnea	Days in Hospital	Days in PICU	Troponin Levels at Admission *	Intervention
1	17	M	1	None	Yes	No	4	0	4006	NSAIDS
2	16.9	M	3	None	Yes	No	4	0	8052	NSAIDS
3	21	M	5	Myocarditis	Yes	No	8	5	2300	NSAIDS
4	16.8	M	2	None	Yes	No	5	3	20,170	NSAIDS
5	17.4	M	4	None	Yes	No	4	2	11,263	NSAIDS
6	17.5	M	1	None	Yes	No	6	5	<2.5	NSAIDS
7	20	M	1	Celiac	No	Yes	4	0	6232	NSAIDS Short hemodynamic support
8	19	M	1	Asthma	Yes	Yes	4	3	13,801	NSAIDS Short hemodynamic support
9	18	M	1	IGA deficiency and myocarditis	Yes	No	1	0	1676	NSAIDS

* Normal value: 0–5 ng/L.

**Table 2:** Clinical and laboratory data for 78 patients with pediatric multisystem inflammatory syndrome (PIMS) and 9 patients with post-COVID vaccination myocarditis in Israel.

Characteristics	Israeli Cohort of Patients with PIMS N = 78	Israeli Post-Vaccination Myocarditis Cohort N = 9
**Demographic characteristics**		
Sex (female)	31 (39.7%)	0 (0%)
Age (years)	9.9 ± 4.5	18.2 ± 1.5
Medical history (positive) *	12 (15.4%)	4 (44.4%)
**Clinical characteristics**		
Shortness of breath at admission	13 (27.3%)	2 (22.2%)
Gastrointestinal symptoms at admission	66 (84.6%)	2 (22.2%)
Mucosal changes at admission	20 (25.6%)	2 (22.2%)
Rash at admission	45 (57.7%)	0 (0%)
Conjunctivitis at admission	31 (39.7%)	0 (0%)
Extremity changes at admission	8 (10.3%)	0 (0%)
Lymphadenopathy at admission only	18 (23.1%)	1 (11%)
CNS involvement at admission	14 (17.9%)	2 (22.2%)
Lung involvement at admission only	6 (7.7%)	0 (0%)
No. of clinical criteria of KD ** w/o fever	1.8	0.3
Fever at admission	77 (98.7%)	5 (55%)
Hypotension	48 (61.5%)	1 (11.1%)
**Cardiac involvement**		
Coronary ectasia (acute)	6 (7.9%)	0 (0%)
Coronary aneurism (acute)	1·(1.3%)	0 (0%)
Left ventricular dysfunction	32 (41.6%)	1 (11.1%)
Coronary brightness	13 (16.9%)	0 (0%)
Pericardial effusion	23 (29.9%)	0 (0%)
Other echo findings	19 (25.0%)	4 (44.4%)
**Laboratory characteristics**		
CRP admission (mg/dL)	18.0 ± 10.4	4.4 ± 3.4
Abnormal BNP ***	38/41 (92.6%)	3/6 (50%)
Troponin	38/71 (53.5%)	8/9 (88.8%)
**Treatment and outcomes**		
Respiratory support	20/78 (25.6%)	0 (0%)
Corticosteroid treatment	69/78 (88.5%)	0 (0%)
**Clinical outcomes**		
Length of hospitalization (days)	8.7 ± 4.7	4.4 ± 1.9
PICU admission	44/78 (56.4%)	6/9 (66.6%)
Length of PICU hospitalization (days)	4.8 ± 3.8	3.6 ± 1.3

* Any chronic disease; ** KD—Kawaski disease; *** BNP.

**Table 3:** Clinical and laboratory data of 69 patients with pediatric multisystem inflammatory syndrome (PIMS) <16 years and nine patients ≥16 years.

Characteristics	Patients with PIMS <16 Years N = 69	Patients with PIMS ≥16 Years N = 9	*p*
**Demographic characteristics**			
Sex (female) (%)	28(40.6)	2(22)	
Age (years)	8.9 ± 3.9	16.7 ± 0.4	
**Clinical characteristics**			
Gastrointestinal symptoms at admission (%)	58(84)	8(88.8)	0.58
Mucosal changes at admission (%)	20(28.9)	1(11.1)	0.35
Rash at admission	40(58)	5(55.5)	0.79
Conjunctivitis at admission	29(42)	2(22)	0.41
Extremity changes at admission	7(10.1)	1(11.1)	1
Lymphadenopathy at admission only	14((20.3)	4(44.4)	0.29
CNS involvement at admission	11(15.6)	3(33.3)	0.55
No. of clinical criteria of KD ** w/o fever	1.86 ± 1.36	1.44 ± 0.88	0.77
Hypotension	42(60.8)	6(66)	0.336
**Cardiac involvement**			
Coronary aneurism (acute)	0	1(11.1)	0.1
Pericardial effusion	20(28.9)	3(33.3)	0.4
**Laboratory characteristics**			
CRP admission (mg/dL)	17 ± 10.2	26.5 ± 6.6	**0.008**
**Treatment and outcomes**			
Respiratory support	15(21.7)	5(55.5)	**0.001**
Hemodynamic support	31(44.9)	6(66)	**0.01**
**Clinical outcomes**			
Length of PICU hospitalization (days) (median max–min)	2(1–9)	3(2–8)	**0.001**

** KD—Kawaski disease.

## Data Availability

Not applicable.
